# Supplementary material for: Comparison of new and old BacT/ALERT aerobic bottles for detection of Candida species
Source: PLoS One. 2023 Nov 29;18(11):e0288674. doi: 10.1371/journal.pone.0288674 (PMC10686453; doi:10.1371/journal.pone.0288674)
Supplement: S1 Table — (DOCX) [file pone.0288674.s001.docx]

**S1 Table. A summary of the *Candida* specimen included in retrospective analysis**

| *Candida* species | Old FA Plus  Number of blood specimen (Peripheral blood specimen) | New FA Plus  Number of blood specimen (Peripheral blood specimen) |
| --- | --- | --- |
| *Candida albicans* | 8(2) | 19(10) |
| *Candida glabrata* | 8(3) | 11(7) |
| *Candida parapsilosis* | 16(6) | 13(8) |
| *Candida tropicalis* | 17(11) | 7(3) |
| Total | 49(22) | 50(28) |
